# Psychosocial Determinants of Mental Health and Risk Behaviours in Adolescents

**DOI:** 10.5539/gjhs.v6n4p22

**Published:** 2014-04-01

**Authors:** M. Carvalho, M. G. Matos, Social Adventure Project Team

**Affiliations:** 1 ISMAT Department of Psychology, Portugal; 2 Mental Health Department of CHBA, Portugal; 3 Centre of Malaria and Tropical Diseases – Associate Laboratory, Portugal; 4 FMH/University of Lisbon, Portugal

**Keywords:** emotional problems, trends, psychosocial determinants, adolescents, gender differences

## Abstract

This study aimed at identifying the prevalence of emotional problems among children and adolescents in Portugal. Gender, developmental aspects, their psychosocial determinants, and the time trends over 8 years were also explored.

The three waves of a cross-sectional survey obtained from the HBSC nationally representative samples of 10-17 year old children and adolescents in 1998, 2002, and 2006, were used. Specific composite indexes included emotional and somatic symptoms, substances’ use, demographic and psychosocial factors.

Girls reported more emotional symptoms, and boys reported more substances’ use. Emotional symptoms and substances’ use increased with age, in contrast school commitment and perception of safe neighbourhood decreased with age. With age, the communication with the family tends to become more difficult, while communication with the friends tends to become easier. Along the three waves, substances’ use and emotional symptoms have shown a general pattern of decrease.

Results were discussed according to literature and their consequences for the understanding of emotional problems and substance use in childhood and adolescence.

Mental health promotion includes both the prevention of emotional problems and risk behaviours; determinants include individual factors and a range of psychosocial factors. Mental health problems have a huge impact on adolescents’ well-being; however it is often a poorer area of intervention in school based interventions. Gender differences are highlighted.

## 1. Introduction

Internalizing and externalizing problems in childhood and adolescence are very common and particularly relevant, due to their impact on psychosocial development. Among emotional and behavioural problems in childhood and adolescence, anxiety, depression, and substance use disorders are referred in literature as the most common (Matos et al., 2012; Ottova et al., 2012; Merikangas et al., 2010; Merikangas, Nakamura, & Kessler, 2009; Kuntsche et al., 2009). Lifetime prevalence rates for depression range from 15 to 20% in clinical samples (Birmaher et al. 1996; Kessler & Walters, 1998; Lewinsohn, Rhode, & Seeley, 1998) and from 22 to 60% in community samples (Roberts et al., 1990; Kubik et al., 2003), whereas lifetime prevalence rates for anxiety range from 11 to 17% in community samples, and from 27 to 45% in clinical samples (Essau, Conradt, & Peterman, 2000; Weiss & Last, 2001). Amongst behavioural problems, substances’ use, which is most reported in adolescence, may be a consequence of the maintenance of emotional and behavioural problems (Buckner & Schmidt, 2009; Costello et al., 2003; Kessler et al., 2007; King & Chassin, 2008; Marmorstein, White, Loeber, & Stouthamer-Loeber, 2010; Matos et al., 2010).

Gender and developmental differences in emotional and behavioural problems are also well documented: girls report greater emotional difficulties and boys report more behavioural problems (Crick & Zahn–Waxler, 2003; Schulte, Ramo, & Brown, 2009; Matos et al., 2013; Matos et al., 2012; Cruz et al., 2013); behavioural problems decrease with age and emotional problems increase with age (Bennett, Vora, & Rheingold, 2004; Canino et al., 2004; Costello et al., 2003; Gilliom & Shaw, 2004; Vitaro, Brendgen, & Barker, 2006; Patterson, Degarmo, & Knutson, 2000; Tremblay, 2000; Willoughby, Chalmers, & Busseri, 2004; Matos et al., 2012; Gaspar et al., 2012). Preschool behavioural problems predict later emotional problems (Mesman, Bongers, & Koot, 2001). With age, communication with the family tends to decrease both in quantity and quality, while communication with the friends tends to increase both in quality and quantity (Tomé et al., 2011, 2012).

According to the main theoretical models on emotional and behavioural disorders in childhood and adolescence, different factors are involved on their onset, maintenance, and modification. Several studies carried out in order to identify these factors, have shown that (a) genetic (Sprangers et al., 2010), (b) individual – attachment, temperament, emotional dysregulation, and information processing – (Cisler, Olatunji, Feldner, & Forsyth, 2010; Feng, Shaw, & Silk, 2008; Jansen, Veenstra, Ormel, Verhulst, & Reijneveld, 2011; Mazza, Fleming, Abbott, & Catalano, 2010; Neumann, van Lier, Frijns, Meeus, & Koot, 2011; Tobias, Guiney, Fonagy, Mayes, & Luyten, 2011; van der Zwaluw et al., 2008; Wetter & Hankin, 2009; Matos et al., 2013), (c) family - parental psychopathology and communication – (Copeland, Shanahan, Costello, & Angold, 2009; Cortes, Fleming, Mason, & Catalano, 2009; Feng, et al., 2008; Jaser, et al., 2008; Knappe, Beesdo, Fehm, Lieb, & Wittchen, 2009; Piche, Bergeron, Cyr, & Berthiaume, 2011; van Oort, Verhulst, Ormel, & Huizink, 2010; Warner, Wickramaratne, & Weissman, 2008; Tomé et al., 2011; 2012), and (d) social/contextual variables - peers pressure, school, neighbourhood, and life events- (Carter et al., 2010; Hankin, 2008; McLaughlin & Hatzenbuehler, 2009a; McLaughlin & Hatzenbuehler, 2009b; McLaughlin et al., 2010; Matos et al., 2010; Matos et al., 2012), are related to emotional and behavioral problems. However, only a small number of studies specifically considers family, social and contextual factors and, even scarcer, only a few analyse their developmental aspects.

The goals of the present study were threefold: a) to analyse gender and grade differences for individual, family and school variables in the three waves of the nationally representative sample of the HBSC between 1998 and 2006, b) to analyse the time trends of emotional problems, substance use and related family and school factors, and c) to understand the individual, familial, and school predictors of emotional problems and substance use.

## 2. Method

### 2.1 Participants

The three waves of the Portuguese sample of the HBSC include 17911 adolescents, 47% male and 53% female, aged between 10 and 17 years old (mean age of 14 years old), in the 6^th^ (35.3%), 8^th^ (36.3%), and in the 10^th^ school year (28.3%), randomly assigned from national schools and stratified, representing all the country.

In 1998, the first Portuguese sample was composed of 6903 children and adolescents, 53% females and 47% males, with a mean age of 14 years old (Matos & Social Adventure Team, 2000). In 2002, the second sample was composed of 6131 children and adolescents, 49% males and 51% females, also with a mean age of 14 years old (Matos, & Social Adventure Team, 2003). Finally, in 2006, 4877 children and adolescents composed the third sample, 50.4% females and 49.6% males (Matos et al., 2006). Table 1 shows the demographic characteristics of the three samples, according to the database year. Details on the other demographic characteristics of the three samples can be found in Matos and Social Adventure Team (2000, 2003), and in Matos and colleagues (2006).

**Table 1:** Demographic characteristics of the sample according to the database year

	1998		2002		2006	
		
	N	%	N	%	N	%
Gender						
Male	3241	47	2417	49.6	3006	49
Female	3662	53	2460	50.5	3125	50
School Grade						
6^th^ grade	2409	31.7	1546	31.7	2369	38.6
8^th^ grade	2589	37.5	1740	35.7	2181	35.6
10^th^ grade	1905	27.6	1591	32.6	1581	25.8
Age	14.12	1,71	14	1.85	14.05	1.89

### 2.2 Measures

The questionnaire relied upon (Currie et al., 2004) composed of two parts. The main part includes a demographic data section and the assessment of school environment, alcohol and tobacco consumption, violence, physical activity and hobbies, nutrition, security, psychosocial health, general symptoms, social relationships and social support. In the second part, questions about drugs consumption and HIV information, attitudes and behaviours were included. Each questionnaire requires about 55 minutes to be administered. In the present study, several composite indexes were computed, in order to assess the main individual (emotional problems and substances’ use), family, peer (communication), and contextual factors (commitment to school and safe neighbourhood): school commitment was assessed by the sum of the five items related to school factors; emotional problems were assessed by the sum of the participants’ responses to the 10 items assessing somatic symptoms and nervousness and sadness; substances’ use was computed from the sum of the four items assessing smoking, alcohol and drugs consumption; communication with significant others, family and friends, was assessed by the sum of the responses to the 11 relevant items, and, finally, safe neighbourhood was computed from the nine items relevant to assess the perception of a safe neighbourhood (see Table 2).

**Table 2:** Items used and range

Items	Range
School commitment	
School performance	1 – 4 (very good/below average)*
Liking school	1 -4 (a lot/not at all)*
School mates like being together	1 – 5 (always true/always false)*
School mates acceptance	1 – 5 (always true/always false)*
Homework pressure	1 – 4 (none/a lot)*
Somatic symptoms	
Headaches	1 -5 (almost every day/almost never or never)*
Stomac aches	1 -5 (almost every day/almost never or never)*
Nervousness and sadness	
Sad/depressed	1 -5 (almost every day/almost never or never)*
Angry/bad mood	1 -5 (almost every day/almost never or never)*
Nervous	1 -5 (almost every day/almost never or never)*
Substances’ use	
Smoking	1 – 4 (every day/don’t smoke)*
Drinking	1 – 5 (every day/never)*
Intoxicated	1 – 5 (never/more than 10 times)
Drugs	1 – 4 (none/regularly)
Communication with family	
At ease speaking with father	1 – 4 (very easy/very difficult)*
At ease speaking with mother	1 – 4 (very easy/very difficult)*
At ease speaking with older brother	1 – 4 (very easy/very difficult)*
At ease speaking with older sister	1 – 4 (very easy/very difficult)*
Communication with friends	
At ease speaking with best friend	1 – 4 (very easy/very difficult)*
At ease speaking with same sex friend	1 – 4 (very easy/very difficult)*
At ease speaking with other sex friend	1 – 4 (very easy/very difficult)*
Safe neighbourhood	
Get along weel	0 – 1 (no/yes)
Safe place	0 – 1 (no/yes)
Trust persons	0 – 1 (no/yes)
Hobbies	0 – 1 (no/yes)
Night fun	0 – 1 (no/yes)*
Violence/robery	0 – 1 (no/yes)*
Nice	0 – 1 (no/yes)
Too withdrawn	0 – 1 (no/yes)*
Good public services	0 – 1 (no/yes)

* reverted items.

### 2.3 Procedure

The schools that took part on the sampling process were randomly selected from the national schools list, and stratified by educational regions. In each school, a random selection of classes was carried out and the questionnaire was administered by the teachers in the classroom, in group, after the students’ informed consent to their volunteer and anonymous participation in the study. Details on the procedures for data collection in the HBSC Study can be consulted in Matos and Social Adventure Team (2000, 2003), and in Matos and colleagues (2006).

### 2.4 Statistical Analysis

SPSS 18.0 for Windows (SPSS, Chicago IL, USA) was used in order to carry out Qui-square, t-Student and One-way ANOVAs tests to compare groups. Multiple linear regressions were carried out to study the associations between the variables.

## 3. Results

### 3.1 Descriptive Data for Individual, Family, and School Composite Indexes

Table 3 shows the descriptive data obtained for all the indexes composed to assess emotional problems, substances’ use, communication, and safe neighbourhood. Except for somatic symptoms and substances’ use, all skewness values were similar to the normal curve; kurtosis values on those variables, as for communication with significant others, also did not assume normality. However, due to the sample size, parametric statistics were used in further analyses.

**Table 3:** Descriptive data for individual, family, and school composite indexes

	Number of items	M	SD	Range	Skewness	Kurtosis
School commitment	5	16.72	2.35	5-22	-.51	.62
Emotional Symptoms	5	9.34	.408	5-25	1.05	.60
Somatic symptoms	2	3.24	1.74	2-10	1.51	1.82
Nervousness and sadness	3	6.11	3.05	3-15	.96	.11
Substances’ use	4	5.44	2.31	4-18	2.32	5.83
Communication with significant others	7	24.06	3.91	7-35	-.66	1.21
Family	4	14.33	2.82	4-20	-.93	1.07
Friends	3	9.69	2.08	3-15	-.56	.69
Safe neighbourhood	9	12.21	1.87	7-18	-.33	1.03

### 3.2 Time Trends of Substances’ Use

The evolution of substances’ use was analysed along the three waves (see Table 4). Significant associations between the database year and smoking consumption, *χ^2^* (6) = 127.36, *p* = .0001, alcohol consumption, *χ^2^* (8) = 971.97, *p* = .0001, drunkenness, *χ^2^* (8) = 44.69, *p* = .0001, and drugs consumption, *χ^2^* (6) = 158.17, p = .0001, were found. Although most of the participants did not report consumptions, an increase of smoking consumption, regular drinking and drugs consumption was found in 2002. The increase in regular drugs consumption was maintained in 2006. A different pattern was identified for the number of intoxication episodes: although, again, most of the participants reported not having intoxication episodes, the adolescents tend to report a greater amount of intoxication episodes over time, from 1998 to 2006.

**Table 4:** Substances’ use according to the database year

	1998 (N = 6561)		2002 (N = 5921)		2006 (N = 4713)		χ^2^
		
	N	%	N	%	N	%	
Smoking consumption							127,36***
I don’t smoke	**5915**	**86.9**	4943	81.4	**4212**	**87.8**	
Less than once a week	339	5	**340**	**5.6**	211	4.4	
At least once a week	185	2.7	**274**	**4.5**	132	2.8	
Everyday	368	5.4	**513**	**8.5**	240	5	
Alcohol consumption							971,97***
Never	2788	42.6	**3829**	**63.7**	**2999**	**62.6**	
Rarely	**3109**	**47.5**	1447	24.1	1257	26.2	
Every month	433	6.6	394	6.6	323	6.7	
Every week	195	3	**283**	**4.7**	179	3.7	
Everyday	26	.4	**60**	**1**	33	.7	
Intoxicated							44.69***
Never	**5356**	**77.9**	4562	75.5	3552	73.7	
Once	763	11.1	715	11.8	553	11.5	
Two or three times	471	6.8	445	7.4	**425**	**8.8**	
Four to ten times	140	2	173	2.9	155	3.2	
More than ten times	**148**	**2.2**	150	2.5	134	2,8	
Drugs consumption (one month)							158.17***
Never	**6155**	**97.5**	5227	93.4	4237	95.5	
Once	66	1	**132**	**2.4**	86	1.9	
More than once	90	1	**152**	**2.7**	66	1,5	
Regularly	0	0	**86**	**1.5**	**48**	**1.1**	

Note:

*** *p* < .001. *Adjusted residuals superior to 1.9 are shown in bold*.

### 3.3 Gender comparisons for individual, family, peers and contextual factors

Gender comparisons have shown significant differences for the total score of emotional symptoms, *t* (17103) = -28.95, *p* = .0001, somatic symptoms, *t* (17448) = -30.26, *p* = .0001, nervousness and sadness, *t* (17203) = -21.54, *p* = .0001, and substances’ use, *t* (15686) = 12.25, *p* = .0001; girls reported more emotional symptoms and boys reported more substances’ use. Significant gender differences were also found for the total score of communication with significant others, *t* (7357) = 6.03, *p* = .0001, and specifically for communication with family, *t* (13095) = 7.90, *p* = .0001, and friends, *t* (9773) = 2.98, *p* = .003, with boys reporting being more at ease when communication with significant others. Finally, a gender difference was also found for safe neighbourhood, *t* (4354) = -9.02, *p* = .0001, with girls reporting perceptions of a more safe neighbourhood than boys. No gender differences were found for school commitment, p > .05 (see Table 5).

**Table 5:** Gender comparisons for individual, family, peers and contextual factors

	Males (N = 8265)		Females (N = 8930)		t	η^2^
	
	M	SD	M	SD		
Emotional symptoms	8,42	3,61	10,18	4,30	-28,95***	.082
Somatic symptoms	2,84	1,47	3,61	1,88	-30,26***	.083
Nervousness and sadness	5,59	2,81	6,58	3,19	-21,54***	.069
Substances’ use	5.68	2.55	5.22	2.05	12.25***	.055
Communication						
Significant others	24,36	4,14	23,81	3,68	6,03***	.057
Family	14,54	2,83	14,15	2,80	7,90***	.049
Friends	9,76	2,22	9,63	1,95	2,98**	.034
School commitment	16,74	2,33	16,69	2,37	1.50	.018
Safe neighbourhood	11,95	1,96	12,46	1,75	-9,02***	.209

Note:

** *p* ≤ .01;

*** *p* ≤ .001.

### 3.4 Grade Comparisons for Individual, Family, Peers and Contextual Factors

Developmental comparisons, based on school grade, have shown significant differences in emotional symptoms, *F* (2; 17102) = 176.33, *p* = .0001, and specifically in somatic symptoms, *F* (2; 17447) = 70.48, *p* = .0001, nervousness and sadness, *F* (2; 17202) = 168.11, *p* = .0001, and, also, in substances’ use, *F* (2; 15685) = 999.75, *p* = .0001. Participants in the 6^th^ grade reported less emotional and behavioural symptoms compared to participants in the 8^th^ grade, which also reported fewer symptoms than participants in the 10^th^ grade. Grade differences were also found for communication with significant others, *F* (2; 7356) = 9.23, *p* = .0001, and specifically with family, *F* (2; 13094) = 40.38, *p* = .0001, and friends, *F* (2; 9772) = 34.23, *p* = .0001, with 8^th^ graders reporting being more at ease communicating with significant others. However, when specifically considered, 6^th^ graders reported being more at ease communicating with their families, compared to 8^th^ and 10^th^ graders, and less at ease communicating with friends compared to participants in the 8^th^ grade, which also reported being less at ease than 10^th^ graders when communicating with friends. School commitment and safe neighbourhood were also significantly different according to school grade, *F* (2; 17192) = 361.44, *p* = .0001, and *F* (2; 4353) = 32.32, *p* = .0001, respectively: 6^th^ graders reported less school commitment than 8^th^ graders, which reported less school commitment than 10^th^ graders; 6^th^ graders reported a perception of a less safe neighbourhood compared to 8^th^ and 10^th^ graders (see Table 6).

**Table 6:** Grade comparisons for individual, family, peers and contextual factors

	6^th^ Grade (a) (N = 6324)		8^th^ Grade (b) (N=6510)		10^th^ Grade (c) (N=5077)		F
		
	M	SD	M	SD	M	SD	
Emotional symptoms	8.63	3.84	9.4	4.0	10.09	4.22	176.33*** a<b<c
Somatic symptoms	3.08	1.69	3.22	1.71	3.47	1.79	70.48*** a<b<c
Nervousness/sadness	5.57	2.90	6.22	3.09	6.62	3.09	168.11*** a<b<c
Substances’ use	4.56	1.32	5.42	2.22	6.51	2.83	999.75*** a<b<c
Communication							
Significant others	24.22	4.15	23.08	4.01	24.19	3.48	9.23*** a>b, c
Family	14.64	2.86	14.13	2.90	14.23	2.65	40.38*** a>b, c
Friends	9.50	2.30	9.67	2.07	9.93	1.82	34.23*** a<b<c
School commitment	17.3	2.39	16.64	2.3	16.11	2.2	361.44*** a>b>c
Safe neighbourhood	11.88	1.94	12.28	1.88	12.43	1.76	32.32*** a<b, c

Note:

*** *p* ≤ .001.

### 3.5 Time Trends in Individual, Family, Peers and Contextual Factors

Individual, family, peer and contextual factors evolution was studied according to gender (except for school commitment which showed no gender differences). Univariate ANOVA performed for school commitment showed a significant difference, *F* (2; 17192) = 276.01, *p* = .000, with 6^th^ graders reporting less school commitment than 8^th^ graders, which reported less school commitment than 10^th^ graders (see Table 7).

**Table 7:** Database year comparisons for school factors

	1998 (a) (N = 6561)		2002 (b) (N = 5921)		2006 (c) (N = 4713)		F
		
	M	SD	M	SD	M	SD	
School commitment	17,24	2,21	16,47	2,42	16,29	2,34	276,01*** a>b>c

Note:

*** *p* ≤ .001.

Univariate ANOVAS 3*2 (database * gender) were performed for all the other variables. When main effects were significant, Post-hoc Scheffé tests, based on inspection of the sub-class means presented in Table 8, were undertaken to understand the nature of the interaction.

**Table 8:** Database, year and gender comparisons for individual, family, peers and contextual factors

	1998				2002				2006				F y	F y*g	F g

	M		F		M		F		M		F				

	M	SD	M	SD	M	SD	M	SD	M	SD	M	SD			
Emotional symptoms	8,6	3,6	10,2	4,3	8,6	3,6	10,5	4,3	7,8	3,4	9,6	4,2	67,30***	3,59*	821,35***
Somatic symptoms	3,0	1,5	3,7	1,9	2,7	1,4	3,5	1,8	2,7	1,3	3,4	1,8	62,48***	1,34	879,49***
Nervousness and sadness	5.6	2.7	6.4	3.1	5.9	2.9	7	3.2	5.1	2.6	6.2	3.1	89,28**	3,85***	462,54*
Substances’ use	5.6	2.3	5.2	1.7	5.7	2.8	5.2	2.2	5.5	2.4	5.1	2.1	120.27***	61.78***	6.77***
Communication															
Significant others	-	-	-	-	8,6	3,6	10,5	4,3	8.6	3.6	10.2	4.3	30.1**	1.3	36.2**
Family	14.3	2.8	14	2.8	14.7	2.7	14.3	2.7	14.5	2.9	14	2.8	14.7**	1.2	65.2**
Friends	-	-	-	-	9.9	2.1	9.6	1.9	9.5	2.3	9.5	1.9	27.3**	7.5***	7.3***

Note: M = Male; F = Female; y = year; g = gender.

* *p* ≤ .05;

** *p* ≤ .01;

*** *p* ≤ .001.

A significant main effect of database year, *F* (2) = 67.30, *p* = .0001, *η^2^* = .008, and gender, *F* (1) = 831.35, *p* = .0001, *η^2^* = .046, qualified by a significant Database × Gender interaction, *F* (2) = 3.59, *p* = .027, *η^2^* = .000, was found for emotional symptoms. Post-hoc tests to follow up the main effect of database, revealed that in 2006, less emotional symptoms were reported, compared to 2002 and 1998, and that girls reported more emotional symptoms than boys. For somatic symptoms, a significant main effect of database year, *F* (2) = 62.48, *p* = .0001, *η^2^* = .007, and gender, *F* (1) = 879.49, *p* = .0001, *η^2^* = .048, were also found. The Database × Gender interaction effect was not significant, *p* > .05. Post-hoc tests to follow up the main effect of database, revealed that in 1998, more somatic symptoms were reported, compared to 2002 and 2006, and that girls reported more somatic symptoms than boys. Finally, for nervousness and sadness, a significant main effect of database year, *F* (2) = 89.28, *p* = .0001, *η^2^* = .010, and gender, *F* (1) = 362.54, *p* = .0001, *η^2^* = .026, qualified by a significant Database × Gender interaction, *F* (2) = 3.85, *p* = .021, *η^2^* = .000, was found. Post-hoc tests to follow up the main effect of database revealed that, in 2002, participants reported more nervousness and sadness compared to 1998, which, in turn, reported more somatic symptoms than in 2006. Following the previous pattern, girls reported more nervousness and sadness than boys.

For substances’ use, a significant main effect of database year, *F* (2) = 120.27, *p* = .0001, *η^2^* = .015, and gender, *F* (1) = 61.78, *p* = .0001, *η^2^* = .004, qualified by a significant Database × Gender interaction, *F* (2) = 6.77, *p* = .001, *η^2^* = .001, was found. Post-hoc tests to follow up the main effect of database revealed that participants reported using more substances’ in 2002 compared to 2006, and that boys reported more substances’ use than girls.

When assessing communication, a significant main effect of database year, *F* (2) = 30.14, *p* = .0001, *η^2^* = .001, and gender, *F* (1) = 36.20, *p* = .0001, *η^2^* = .005, were found. The Database × Gender interaction effect was not significant, *p* > .05. Post-hoc tests to follow up the main effect of database revealed that participants reported being more at ease when communicating with significant others in 2002 compared to 2006, and that boys reported being more at ease than girls. Specifically, a significant main effect of database year, *F* (2) = 14.75, *p* = .0001, *η^2^* = .002, and gender, *F* (1) = 65.29, *p* = .0001, *η^2^* = .005, was found for communication with family, and a significant main effect of database year, *F* (2) = 27.37, *p* = .0001, *η^2^* = .003, and gender, *F* (1) = 7,36, *p* = .007, *η^2^* = .001, was found for communication with friends. The Database × Gender interaction effect was not significant for communication with family, *p* > .05. However, a significant Database x Gender interaction effect was found for communication with friends, *F* (2) = 7.50, *p* = .006, *η^2^* = .001. Post-hoc tests to follow up the main effect of database, revealed that participants reported being more at ease when communicating with family in 2002 compared to 1998 and 2006, and reported being more at ease when communication with friends in 2002 compared to 2006; in both cases, boys reported being more at ease than girls.

Database x gender interaction effects for emotional and somatic symptoms, nervousness and sadness, and for substances’ use show that emotional problems were higher in 2002, particularly in girls, whereas communication with friends was also higher in 2002 but for boys.

### 3.6 Psychosocial Predictors of Emotional and Problems and Substance Use

Linear multiple regression analysis, using the stepwise method (p<.05), were performed, in order to identify the main factors predicting emotional symptoms and substances’ use (see Table 9).

**Table 9:** Psychosocial predictors of emotional and problems and substance use

IV	Step	DV	R^2^	R^2^ adjusted	β	t

Emotional symptoms	1	School commitment	.101	.100	-.28	-16.04***
	2	Gender	.133	.132	.16	9.42***
	3	Communication with family	.143	.142	-.10	-5.75***
	4	Safe neighbourhood	.144	.143	.03	2.26*
	5	School grade	.146	.144	.03	2.11*
Explained variance				14.4%		

IV	Step	DV	R^2^	R^2^ adjusted	β	t

Substance Use	1	School grade	.107	.106	.27	15.17***
	2	School commitment	.143	.142	-.19	-10.85***
	3	Communication with friends	.160	.159	.16	6.87***
	4	Gender	.166	.165	-.08	-4.62***
	5	Communication with significant others	.168	.166	-.05	-2.06*
Explained variance				16.6%		

Note: IV = Independent variable; DV = Dependent variable;

* *p* ≤ .05;

*** *p* ≤ .001.

For emotional symptoms, a model composed of five independent variables was identified, and explained approximately 14% of total variance: less school commitment, *β* = -.28, *t* = -16.04, *p* = .0001, female gender, *β* = .16, *t* = 9.42, *p* = .0001, less communication with family, *β* = -.10, *t* = -5.75, *p* = .0001, higher perception of a safe neighbourhood, *β* = .03, *t* = 2.26, *p* = .024, and lower school grade, *β* = -28, *t* = -16.04, *p* = .0001, were associated to more emotional symptoms. For substances’ use, a model composed of five independent variables was also identified, explaining approximately 17% of the total variance: higher school grade, *β* = .27, *t* = 15.17, *p* = .0001, less school commitment, *β* = -.19, *t* = -10.85, *p* = .0001, more communication with friends, *β* = .16, *t* = 6.87, *p* = .0001, male gender, *β* = -.08, *t* = -4.62, *p* = .0001, and less communication with significant others, *β* = -.05, *t* = -2.06, *p* = .039, were associated to more substances’ use.

## 4. Discussion

The present study focused on gender and grade differences in individual, family and school variables in the three waves of the nationally representative sample of the HBSC between 1998 and 2006 and intended to analyse the time trends of emotional problems, substance use and related family and school factors as predictors of emotional problems and substance use.

Although its limitations (namely related to the cross sectional nature of the study, to the nature of the sample (non-clinical), and to the fact that only addressed a set of all the main variables that are, according to the literature, relevant for explaining emotional and behavioural problems), these findings may present important implications for the development of prevention and intervention programs, according to grade and gender in order to address specific needs and lighten up their efficacy.

The increase of smoking consumption, regular drinking and drugs consumption in 2002, followed by a subsequent decrease, except for regular drugs consumption, evidences a pattern similar to the pattern obtained in another countries (Kuntsche et al., 2009).

Gender differences found were similar to the literature, with girls reporting more emotional symptoms and boys reporting more substances’ use (Crick & Zahn-Waxler, 2003; Gilliom & Shaw, 2004; Gaspar et al., 2012; Matos, Tomé et al., 2012). Gender comparisons for communication with significant others and perception of a safe neighbourhood were also according to literature (Luk, Farhat, Iannotti, & Simons-Morton, 2010) that is, girls are more sensitive to contextual factors and, although being more “verbal” (Matos et al., 2003) they have the perception of having a poorer interpersonal communication. Other studies using a qualitative approach suggest that girls use interpersonal communication to establish and maintain a close emotional relationship, whereas boys use interpersonal communication in a more instrumental way, to “get things done”, therefore being easier to refer to it as easy, girls tending to be more demanding in relation to what a “good communication” really is (Matos, Simões et al., 2010; Matos, Morgan et al., 2013; Matos, Gaspar et al., 2012, 2013).

Also, developmental differences on emotional and behavioural symptoms, communication with significant others, and school commitment were supported by Luk et al. (2010): along adolescence, emotional and behaviour problems tend to increase (Matos et al., 2000, 2003, 2006, 2008), the quantity and quality of the communication with family tends to decrease, while increasing regarding the peer group (Tomé et al., 2011, 2012).

The new technologies of information and communication, with a special mention to the social networks provided a new insight into interpersonal communication, either with strangers or with significant others. This discussion is far beyond the scope of the present work, but provoked a change in the communication patterns among adolescents (Matos & Ferreira, 2013) that now goes far beyond the communication in “presence”, and is possible even during the time previously devoted to family life.

Finally, the analysis of the psychosocial predictors of emotional problems and substances’ use has shown a set of common factors, school commitment, school grade and gender, and a set of specific factors, communication with family and perception of a safe neighbourhood (regarding emotional symptoms) and communication with friends and, in general, with significant others (regarding substances’ use). These results are similar to the literature (e.g., Luk, et al., 2010; Matos, Simões et al., 2010; Matos, Gaspar et al., 2013) showing, the importance of the interactions between individual and social factors to emotional and behavioral problems.

Mental health problems have a huge impact on adolescents’ well-being, although girls and boys appear to experience environmental constrains and stressful experiences in different ways and they seem to benefit from different protective factors.

Mental health it is a poorer area of intervention in school based health promotion interventions. A new approach to health promotion in Portuguese Schools highlighted the importance of having schools embrace mental health as a major focus, together with sexual and reproductive health, substance use, nutrition, active leisure and interpersonal violence (Matos et al., 2008; Matos, Sampaio et al., 2013) however, except when focusing at very specific social risk contexts (Matos, Gaspar et al. 2012), mental health promotion seems to remain the health promotion “taboo”; this fact has pervasive effects on children and adolescent’s wellbeing, once internalizing and externalizing problems in childhood and adolescence are particularly common and particularly relevant, exactly due to their impact on psychosocial development.

Promoting wellbeing and mental health along childhood and adolescence is not simply a matter of avoiding problematic contexts and health compromising behaviours, recent studies evidenced that increasing social and personal competences (such as self-regulation, negotiating, interpersonal communication and problem solving), and providing social support from significant others can be the golden standard for the promotion of wellbeing and mental health along childhood and adolescence (e.g. Matos et al., 2008; Morgan et al., 2010; Matos, Gaspar et al., 2012; Matos, Morgan, 2012; Matos, Sampaio et al., 2013), recommending a positive focus (the health assets) within a global social context.

Finally, other recent policy roadmap (Ottava et al., 2013) raised the question of children and adolescents participation in the design and implementation of interventions targeting themselves. This last issue “making children voices and lives more visible and happier”, become a scientific-policy motto, deserving further empirical and theoretical validation.

## 5. Conclusion

Mental health promotion includes both the prevention of emotional problems and risk behaviours, and its determinants include individual factors and a range of psychosocial determinants whose identification has a major importance for intervention either universal prevention, selective prevention or indicative prevention, that is in clinical as well as in population interventions.

Mental health problems have a huge impact on adolescents well-being, although girls and boys appear to experience environmental constrains and stressful experiences in different ways, and seem to be differently affected by protective factors. However mental health is often a poorer area in school based interventions.

Gender differences should be considered in future research, as it is necessary to understand in what way there are (still) strong cultural issues, but also if there are biological issues including a different brain maturation that can be related to the gender differences that were highlighted.

Considering the communication with family and peers, the recent boom of social networks and internet-based communication deserves a close follow-up, in order to highlight its positive and negative effects on interpersonal communication along adolescence.

Future studies should empirically test the interactions between individual, social and contextual factors involved in the onset and maintenance of emotional and behavioural problems, using longitudinal designs in order to properly address both developmental issues and the determinants of the onset and maintenance of emotional and behavioural problems.

Empirical and theoretical evidence is also recommended in order to evaluate the relevance and efficacy of the inclusion of children and adolescents as partners, defining own needs and being involved in the design of health promoting programs targeting themselves.
